# Developing a Patient-Reported Outcome Measure for Radionuclide Therapy for Prostate Cancer

**DOI:** 10.2967/jnumed.122.264946

**Published:** 2023-06

**Authors:** Lisa M. Gudenkauf, Melody N. Chavez, Melinda Leigh Maconi, Carley Geiss, Ameen Seyedroudbari, Pan Thin, Aasha I. Hoogland, Kathleen Nguyen, Vishnu Murthy, Wesley R. Armstrong, Khaled Komrokji, Laura B. Oswald, Heather S.L. Jim, Ghassan El-Haddad, Wolfgang P. Fendler, Ken Herrmann, David Cella, Johannes Czernin, Michael S. Hofman, Adam P. Dicker, Jeremie Calais, Scott T. Tagawa, Brian D. Gonzalez

**Affiliations:** 1 Department of Health Outcomes and Behavior, Moffitt Cancer Center, Tampa, Florida;; 2 Participant Research, Interventions, and Measurements Core, Moffitt Cancer Center, Tampa, Florida;; 3 Department of Molecular and Medical Pharmacology, UCLA, Los Angeles, California;; 4 Department of Diagnostic Imaging and Interventional Radiology, Moffitt Cancer Center, Tampa, Florida;; 5 Department of Nuclear Medicine, University of Duisburg–Essen, and German Cancer Consortium–University Hospital Essen, Essen, Germany;; 6 Departments of Medical Social Sciences and Psychiatry and Behavioral Sciences, and Lurie Comprehensive Cancer Center, Northwestern University Feinberg School of Medicine, Chicago, Illinois;; 7 Prostate Cancer Theranostics and Imaging Centre of Excellence, Cancer Imaging, Peter MacCallum Centre, Melbourne, Victoria, Australia, and MacCallum Department of Oncology, University of Melbourne, Melbourne, Victoria, Australia;; 8 Department of Pharmacology and Experimental Therapeutics, Sidney Kimmel Cancer, Thomas Jefferson University, Woodbury, New Jersey; and; 9 Department of Urology, Weill Cornell Medical College, New York, New York

**Keywords:** genitourinary oncology, radionuclide therapy, radiopharmaceuticals, patient-reported outcomes, prostate cancer, radionuclide therapy

## Abstract

The field of radionuclide therapy (RNT) for prostate cancer (PC) is growing rapidly, with recent Food and Drug Administration approval of the first ^177^Lu-PSMA ligand. We aimed to develop the first patient-reported outcome (PRO) measure for PC patients receiving RNT. **Methods:** We identified relevant symptoms and toxicities by reviewing published trials and interviews with PC patients receiving RNT (*n* = 29), caregivers (*n* = 14), and clinicians (*n* = 11). Second, we selected items for measure inclusion. Third, we refined the item list with input from experts in RNTs and PROs. Fourth, we finalized the Functional Assessment of Cancer Therapy–Radionuclide Therapy (FACT-RNT) with patient input. **Results:** This multistep process yielded a brief 15-item measure deemed by key stakeholders to be relevant and useful in the context of RNT for PC. **Conclusion:** The FACT-RNT is a new standardized tool to monitor relevant symptoms and toxicities among PC patients in RNT trials and real-world settings.

## Visual Abstract

**Figure:**
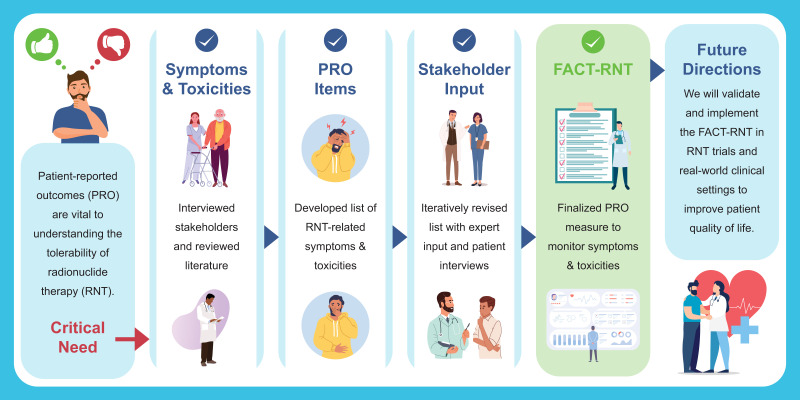

Radionuclide therapy (RNT) is a rapidly emerging class of oncology agents for metastatic castration-resistant prostate cancer (PC), spurred by Food and Drug Administration approval of ^223^Ra-dichloride and ^177^Lu-PSMA-617. RNTs, such as ^177^Lu-PSMA-617, improve radiographic progression-free survival and overall survival versus standard care (1), with a higher biochemical response rate, fewer grade 3 or 4 adverse events than cabazitaxel (2), and improved or preserved health-related quality of life (3*,*^4^).

The Food and Drug Administration encourages patient-reported outcomes (PROs) as a trial primary endpoint (5) or as a complement to clinical and physiologic endpoints (6). Because clinicians may underestimate patient-reported toxicities (7), PROs are crucial to assessing treatment tolerability. Health-related quality of life among RNT recipients was better than among placebo recipients (e.g., ALSYMPCA trial (8)) and similar to that among cabazitaxel recipients (e.g., TheraP trial (2)).

PROs are associated with clinical outcomes, such as improved health-related quality of life among patients with better ^177^Lu-PSMA-617 biochemical response (9). Phase 1 trials (*n* = 79) of ^177^Lu-PSMA-617 or ^225^Ac-J591 showed that RNT response was associated with PRO changes (10). PRO importance is underscored by recent evidence that PRO monitoring improved post-chemotherapy survival and other important outcomes over usual care (11*,*^12^).

Commonly used PRO measures (e.g., EORTC QLQ-C30 (13) and FACT-P (14)) are lengthy and were designed to assess the impacts of conventional therapies (e.g., chemotherapy and radiation). Brief PRO measures designed for RNT are needed to optimize measurement, prognostic value, and cross-trial comparison. This study aimed to develop a brief, targeted PRO measure for PC patients receiving RNT. We hypothesized that a multistep approach to identifying relevant symptoms and toxicities and iterative refinement would yield a brief measure relevant to RNT recipients and experts in the fields of RNT and PROs.

## MATERIALS AND METHODS

**FIGURE 1.**
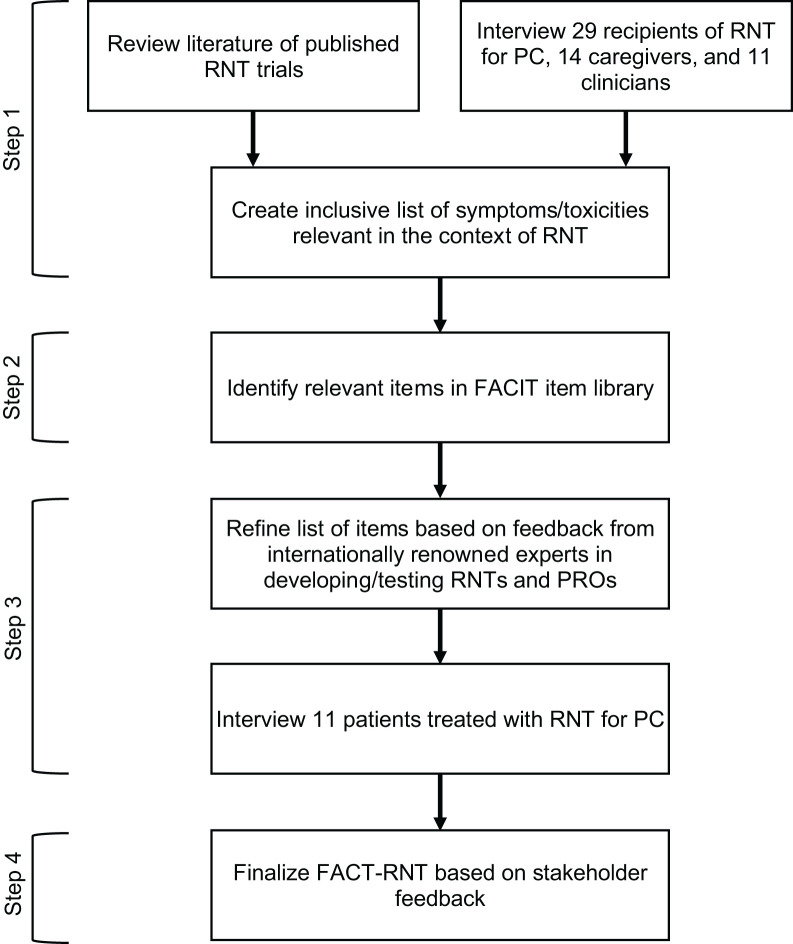
Developing PRO measure for RNT for PC.

Figure 1 shows the study flow for Functional Assessment of Cancer Therapy–Radionuclide Therapy (FACT-RNT) development from 2021 to 2022, following an approach similar to that of other studies developing PRO instruments for cancer patients (15). The protocol was deemed exempt from institutional review board review, and participants provided informed consent verbally. We recruited English- or Spanish-speaking adults, including PC patients who received RNT, informal caregivers (e.g., a spouse or relative) of RNT recipients with PC, and RNT-experienced clinicians at UCLA or Moffitt Cancer Center. Following qualitative research guidelines (16*,*^17^), we aimed to interview at least 10 participants per group until reaching saturation.

This study was conducted in 4 steps. In step 1, we identified an intentionally broad, comprehensive list of RNT symptoms and toxicities (e.g., hematotoxicity and nephrotoxicity) experienced by PC patients during or after RNT. This list was compiled by reviewing published trials and by performing semi-structured interviews with patients, caregivers, and clinicians to elicit common, distressing, and/or clinically meaningful symptoms and toxicities. Each participant was compensated $25. The interviews were audio-recorded, transcribed verbatim, and analyzed with NVivo software, version 12, using the immersion/crystallization method by 2 qualitative research staff with strong interrater reliability (κ ≥ 0.80) until no new qualitative themes were identified within each group (17*,*^18^).

In step 2, we searched for RNT symptoms and toxicities identified in step 1 within the Functional Assessment of Chronic Illness Therapy (FACIT) item library (19), a rigorously developed catalog of more than 700 items and 100 validated measures of chronic illness management.

In step 3, we interviewed internationally renowned experts in the fields of RNT and PROs to seek consensus on RNT-relevant symptoms and toxicities and corresponding FACIT items. We iteratively refined the item list based on recommended item additions/deletions and drafted the FACT-RNT.

In step 4, patients from step 1 reviewed the draft FACT-RNT and participated in semi-structured interviews assessing measure acceptability, comprehensibility, RNT relevance, and self-efficacy for completing the measure. The FACT-RNT was subsequently finalized.

## RESULTS

Literature review and interviews with 29 PC RNT recipients, 14 caregivers of RNT recipients, and 11 clinicians identified RNT-relevant symptoms and toxicities (e.g., fatigue, bone pain, xerostomia). Table 1 provides participant characteristics.

**TABLE 1.** Characteristics of Interviewed RNT Recipients, Informal Caregivers, and Clinicians

Characteristic	Step 1			Step 4
	RNT recipients (*n* = 29)	Caregivers (*n* = 14)	Clinicians (*n* = 11)	RNT recipients (*n* = 11)
Age (y)	72 (8)	64 (11)	56 (8)	71 (9)
Male	29 (100%)	0 (0%)	7 (64%)	11 (100%)
Ethnicity				
Hispanic	0 (0%)	0 (0%)	0 (0%)	0 (0%)
Non-Hispanic	26 (90%)	13 (93%)	8 (73%)	9 (82%)
Unknown/not reported	3 (10%)	1 (7%)	3 (27%)	2 (18%)
Race				
White	24 (83%)	11 (79%)	6 (55%)	8 (73%)
Black/African American	0 (0%)	0 (0%)	0 (0%)	0 (0%)
Asian	1 (3%)	2 (14%)	2 (18%)	0 (0%)
Native American/Alaska Native	0 (0%)	0 (0%)	0 (0%)	0 (0%)
Hawaiian/Pacific Islander	0 (0%)	0 (0%)	0 (0%)	0 (0%)
Unknown/not reported	4 (14%)	1 (7%)	3 (27%)	3 (27%)
Years since diagnosis	11.72 (7.02)			11.84 (7.74)
RNT injections received	4.00 (1.65)			4.45 (1.44)

Categorical data are represented by frequencies and percentages; continuous data are represented by means and standard deviations.

Patients interviewed received ^177^Lu-PSMA-617 and/or ^225^Ac-J591; comprehensive literature review also identified symptoms and toxicities of other RNTs (e.g., ^223^Ra). Notably, interviews identified social isolation as an unexpected and distressing concern due to recommendations to briefly avoid close social contact and public venues post-infusion.

We selected FACIT items corresponding to each symptom and toxicity and to assess functional impacts (e.g., “I am bothered by side-effects of treatment”). In some instances, multiple FACIT items could be used (e.g., “I have a lack of energy” vs. “I feel fatigued”).

Nine RNT and PRO experts from 5 institutions across 3 continents reviewed the draft FACT-RNT and advised on item selection, addition of RNT-relevant symptoms and toxicities (e.g., dry eyes) and removal of less relevant symptoms and toxicities (e.g., neuropathy).

Lastly, 10 RNT recipients from step 1 reported in interviews that FACT-RNT instructions were clear, items were comprehensible and relevant to RNT, response options (e.g., “not at all” vs. “a little bit”) were conceptually distinct, and baseline administration is important to assess changes. Experts and patients recommended adding a bone pain severity item to distinguish among different pain types. Table 2 provides the final FACT-RNT.

**TABLE 2.** FACT-RNT Items

Item code	Symptom or toxicity
HN2	My mouth is dry
ST16	My eyes are dry
P7	I have difficulty urinating
GP2	I have nausea
O2	I have been vomiting
C5	I have diarrhea (diarrhoea)
Pal5	I am constipated
Ga1	I have a loss of appetite
BP1	I have bone pain
HI7	I feel fatigued
AA1	My fatigue keeps me from doing the things I want to do
GP4	I have pain
P3	My pain keeps me from doing things I want to do
GP5	I am bothered by side effects of treatment
Leu7	I feel isolated from others because of my illness or treatment

FACT-RNT is available at FACIT.org/measures/FACT-RNT. Response options range from 1 (not at all) to 5 (very much).

## DISCUSSION

This article describes development of the FACT-RNT, the first (to our knowledge) PRO measure designed for PC patients receiving RNT and developed with multistep feedback from patients, caregivers, clinicians, and experts in RNTs and PROs. FACT-RNT capitalizes on the FACIT item library’s strong validity and reliability, multilanguage translation and validation, and utility as self-administered PROs or via interview.

The FACT-RNT for PC addresses the current gap in measuring RNT-specific symptoms and toxicities and responds to Food and Drug Administration guidance on implementing PROs in therapeutic trials (5) and real-world settings to identify PC patients at risk for deterioration (i.e., worsening symptoms/toxicities). The FACT-RNT was designed for use and future adaptation with a broad variety of RNT agents with different molecular targeting mechanisms and radioisotopes.

The sample was primarily non-Hispanic White; future studies should validate the FACT-RNT in large, diverse prostate cancer samples. Interviews with leading RNT experts helped ensure consideration of symptoms and toxicities relevant to newer-generation RNT agents and ensure the long-term relevance of FACT-RNT items.

## CONCLUSION

We present the FACT-RNT for PC, a new measure developed through multistep collaboration with patients, caregivers, clinicians, and international experts. Next steps include assessment of internal consistency, validity, and reliability and use in RNT clinical trials and real-world settings.

## DISCLOSURE

This work was supported by a grant from the U.S. Department of Defense (W81XWH2010351; principal investigator, Brian Gonzalez) and the Moffitt Cancer Center Participant Research, Interventions, and Measurements (PRISM) Core (P30 CA076292; principal investigator, John Cleveland). Heather Jim reports fees unrelated to this work from consultation for RedHill BioPharma, Janssen Scientific Affairs, and Merck. Ghassan El-Haddad reports consulting fees unrelated to this work from Bayer, Boston Scientific, Canon Medical Systems, Curium Pharma, Novartis, and Terumo. Wolfgang Fendler reports fees unrelated to this work from SOFIE Bioscience, Janssen, Calyx, Bayer, Parexel, and AAA. Ken Herrmann reports personal fees unrelated to this work from Bayer, Sofie Biosciences, SIRTEX, Adacap, Curium, Endocyte, IPSEN, Siemens Healthineers, GE Healthcare, Amgen, Novartis, ymabs, Bain Capital, and Aktis Oncology; grants and personal fees unrelated to this work from BTG; and nonfinancial support unrelated to this work from ABX. David Cella reports that he is president of FACIT.org. Michael Hofman reports grants and fees unrelated to this work from PCF, Peter MacCallum Foundation, Medical Research Future Fund, NHMRC, Movember, and PCFA. Adam Dicker reports advisory activities unrelated to this work with Janssen, Oncohost, Oranomed, CVS, IBA, Aptar (Voluntis), Onconova Therapeutics, and SBRBio. Jeremie Calais reports honoraria unrelated to this work from Astellas, Blue Earth Diagnostics, Curium Pharma, DS Pharma, GE Healthcare, Isoray, IBA RadioPharma, Janssen Pharmaceuticals, Lightpoint Medical, Lantheus, Monrol, Novartis, POINT Biopharma, Radiomedix, Sanofi, and Telix Pharmaceuticals. Brian Gonzalez reports fees unrelated to this work from Sure Med Compliance and Elly Health. No other potential conflict of interest relevant to this article was reported.

## Notes

KEY POINTS **QUESTION:** Can a brief, targeted PRO measure be developed for patients receiving RNT for PC? **PERTINENT FINDINGS:** The FACT-RNT was developed through a multistep, iterative process with input from patients, caregivers, clinicians, and experts in RNT and PROs. **IMPLICATIONS FOR PATIENT CARE:** The FACT-RNT can assess PROs in PC patients receiving RNT.
